# A general chemical transformation route to two-dimensional mesoporous metal selenide nanomaterials by acidification of a ZnSe–amine lamellar hybrid at room temperature†

**DOI:** 10.1039/c6sc00674d

**Published:** 2016-03-09

**Authors:** Zeng-Wen Hu, Liang Xu, Yuan Yang, Hong-Bin Yao, Hong-Wu Zhu, Bi-Cheng Hu, Shu-Hong Yu

**Affiliations:** a Division of Nanomaterials & Chemistry, Hefei National Laboratory for Physical Sciences at the Microscale, Collaborative Innovation Center of Suzhou Nano Science and Technology, CAS Center for Excellence in Nanoscience, Department of Chemistry, Hefei Science Center of CAS, University of Science and Technology of China Hefei 230026 China shyu@ustc.edu.cn

## Abstract

Two-dimensional inorganic nanomaterials have drawn much attention due to their excellent properties and wide applications associated with unique 2D structures. However, an efficient and versatile chemical synthesis method using ambient conditions for 2D nanomaterials, especially with secondary structures (*e.g.* mesopores), has still not been reported. Herein, we report a versatile method to synthesize a family of ultrathin and mesoporous nanosheets of metal selenides based on a precursor so-called “red Se remaining Zn” (RSRZ). The principle of our synthesis is based on a template-assisted chemical transformation process *via* acidification of inorganic–organic hybrid ZnSe(DETA)_0.5_ nanosheets (DETA: diethylenetriamine). An appropriate amount of acid was added into an aqueous dispersion of ZnSe(DETA)_0.5_ nanosheets under air for activation. The acidification induced chemical transformation mechanism was studied by tracking the acidification process. This acid controlled reactivity of lamellar hybrids allows it to be possible to capture the highly reactive intermediates, which will provide a new platform for the synthesis of various mesoporous metal selenides.

## Abstract 2

A family of mesoporous nanosheets of metal selenides can be synthesized using an intermediate precursor so-called “red Se remaining Zn” (RSRZ), which is generated by acidification of inorganic–organic hybrid ZnSe(DETA)_0.5_ nanosheets.

## Graphical abstract

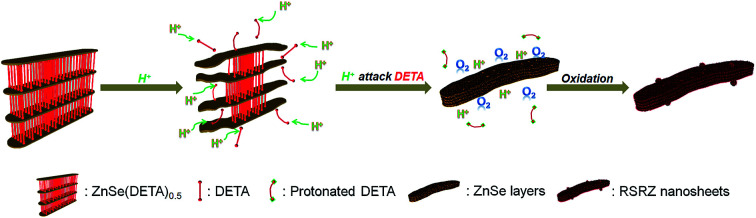

## Introduction

Two-dimensional nanostructural materials have emerged as a new generation of nanomaterials and have been attracting tremendous attention because of their unique properties and wide applications associated with the 2D morphology.^1–9^ Especially, 2D transition-metal chalcogenide nanomaterials such as Cu_*x*_Se nanosheets,^10,11^ CoSe_2_ nanobelts,^12–14^ CdSe nanoplatelets,^15,16^ and MoS_2_ single layers^4,17^ have shown significant potential in various applications, including transistors,^4,18^ lithium-ion batteries,^18^ electrocatalysis^12–14,19^ and so on. The general synthetic strategies to produce 2D nanomaterials can be classified into bottom-up processes and top-down processes. 2D metal–chalcogenide semiconductors were initially prepared *via* the exfoliation of bulk materials. MoS_2_ single layer sheets were exfoliated from commercially available crystals of molybdenite using a scotch-tape micromechanical cleavage technique method that was pioneered for the production of graphene.^4^ A number of metal–chalcogenide nanosheets, such as MoS_2_, WS_2_, MoSe_2_, NbSe_2_, TaSe_2_, NiTe_2_, MoTe_2_, and Bi_2_Te_3_, have been obtained *via* a liquid exfoliation method.^20^ However, the exfoliation method is limited by the structure and components of the bulk material and thus it is very hard to tune the composition and meso-structure of the as-prepared 2D nanomaterials. Fortunately, chemical transformation strategies have also been well developed for the synthesis of 2D nanomaterials, providing the possibility to tune the structure and composition of 2D nanomaterials during the synthesis. Zhang *et al.* have reported that CuSe nanosheets with a microscale lateral size could serve as templates for a phase transformation to synthesize Cu_2−*x*_Se nanosheets.^11^ Kotov *et al.* found that CdTe nanoparticles could self-assemble into free-floating films.^21^ In the chemical transformation process, stabilizers of the nanoparticles play an important role in the assembly. Kotov and Tang have made vital contributions to the development of stabilizer-depletion, including the self-assembly of CdTe nanoparticles into nanowires,^22^ conversion of CdSe nanoparticles into Se nanowires,^23^ and conversion of CdTe nanoparticles into angled Te nanocrystals.^24^ The assembly and chemical transformation of nanoparticles into 2D nanomaterials shows flexibility for materials synthesis but this process is time-consuming and uncontrollable. Recently, the development of a family of metal chalcogenide–amine inorganic–organic hybrid nanomaterials, such as ZnS(DETA)_0.5_ nanobelts,^25^ ZnSe(DETA)_0.5_ nanobelts^26^ and CoSe_2_ hybrid nanobelts,^12^ has exhibited the strong potential of combining chemical synthesis and physical exfoliation together to prepare various 2D metal chalcogenide nanomaterials with tunable compositions and structures.^27–29^ Zhang *et al.* added Cd^2+^ into ZnS(DETA)_0.5_ and ZnSe(DETA)_0.5_ to gain nanoporous Cd_*x*_Zn_1−*x*_S nanosheets and hollow Cd_*x*_Zn_1−*x*_Se nanoframes.^30,31^ However, the details of the efficient chemical conversion of metal chalcogenide–amine inorganic–organic hybrids into 2D metal chalcogenide nanomaterials especially for the stability and reactivity of the hybrid during the conversion process are rarely understood. In addition, the development of a large scale and versatile method to produce metal chalcogenide nanosheets *via* a chemical transformation process remains a great challenge.

Herein, we report a simple and versatile chemical transformation method to prepare ultrathin and mesoporous metal selenide nanosheets starting from ZnSe(DETA)_0.5_ nanosheets using a acidification process. We find that highly reactive nanosheet intermediates called “red Se remaining Zn” (RSRZ) can be prepared by using hydrochloric acid to acidify the ZnSe(DETA)_0.5_ nanosheets, which can act as an excellent template for the chemical transformation reaction. Addition of hydrochloric acid to the amine-assisted hybrid precursors would result in depletion of the amine in the lamellar hybrid structure, followed by dissociation and oxidation of ZnSe. The obtained RSRZ nanosheets can be easily transformed into a family of metal selenide nanostructures including Ag_2_Se nanosheets, Cu_2_Se nanosheets, Pt_*x*_Se_*y*_ alloy nanosheets, Pd_*x*_Se_*y*_ alloy nanosheets, and Se nanowires under ambient conditions.

## Results and discussion

**Scheme 1:**
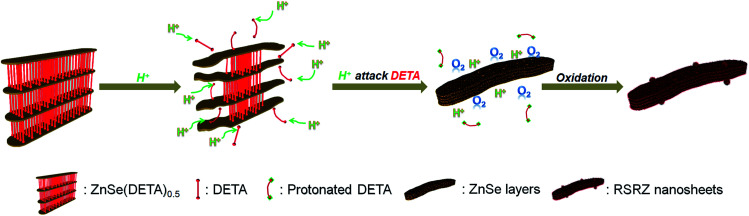
A general illustration of the acidification process of the ZnSe(DETA)_0.5_ lamellar hybrid.

Scheme 1 shows the general acidification strategy process, illustrating the formation of the RSRZ intermediates. H^+^ can diffuse into the lamellar hybrid structure and attack the amine so that the amine will be protonated and released from the hybrid structure. The stabilizer-depleted ZnSe layers are unstable and highly reactive, and are likely to be oxidized to Se by dissolved oxygen in the acidic solution. The standard redox potentials for Se^2−^/Se and O_2_/H_2_O pairs are −0.924 V and 1.229 V, which makes the suggested mechanism thermodynamically possible for the oxidation of Se^2−^,^23^ although the solubility constant (298 K) of ZnSe is as low as 3.6 × 10^−26^.

**Fig. 1:**
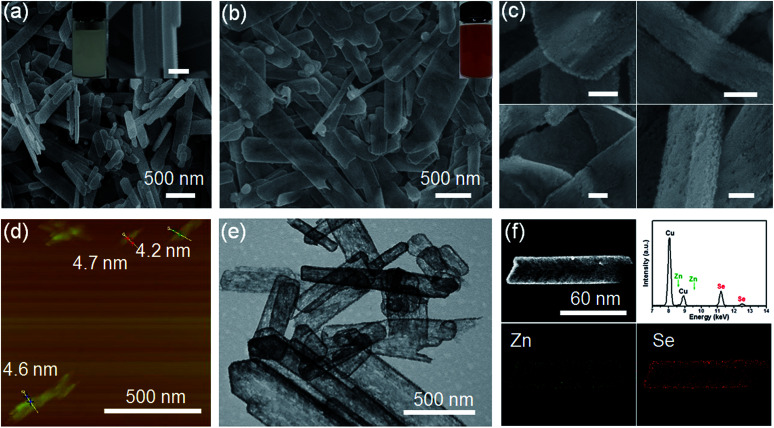
(a) SEM image of ZnSe(DETA)_0.5_ and photograph of the corresponding water dispersion. Inset scale bar is 100 nm. (b) SEM image of the RSRZ nanosheets after acidification for 10 h and a photograph of the corresponding water dispersion. (c) SEM images of the RSRZ nanosheets after acidification for 10 h. Scale bar: 100 nm. (d) AFM image of the RSRZ nanosheets after acidification for 10 h. (e) TEM image of the RSRZ nanosheets after acidification for 10 h. (f) EDS elemental mapping images of the RSRZ nanosheets after acidification for 10 h.

The lamellar structured ZnSe(DETA)_0.5_ hybrids were synthesized as previously reported^26,31^ and used as the starting materials. The as-prepared ZnSe(DETA)_0.5_ nanosheets were examined using scanning electron microscopy (SEM) and X-ray diffraction (XRD). The SEM image (Fig. 1a) showed that the ZnSe(DETA)_0.5_ nanosheets have a thickness of ∼50 nm. The typical XRD pattern (Fig. S1a, ESI†) further confirmed that the hybrid precursor is the same as our reported ZnSe(DETA)_0.5_ nanobelts.^26^ The red floccules precipitated within hours after addition of the hydrochloric acid into the aqueous solution of the ZnSe(DETA)_0.5_ precursor. SEM images (Fig. 1b and c) clearly demonstrated that the size of the RSRZ nanosheets was inherited from the size of the hybrid precursors, except for the thickness which ranged from several nanometers to 30 nanometers. Atomic force microscopy (AFM) images (in Fig. 1d and S1b†) clearly showed that a large part of the RSRZ nanosheets had a thickness of ∼5 nm, indicating that RSRZ nanosheets were successfully exfoliated from the bulk ZnSe(DETA)_0.5_ hybrid with the help of acid. Some incompletely exfoliated nanosheets showed a lamellar structure with a thickness larger than 20 nm (Fig. 1c and S2d, ESI†).

Microscopy characterization clearly showed that the formed RSRZ nanosheets were highly porous (Fig. 1e) and the surface was very rough (Fig. 1c and S1b, ESI†), indicating a potentially large specific surface area. Nitrogen adsorption–desorption isotherms of the RSRZ nanosheets are shown in Fig. S1c (ESI†), which revealed that the RSRZ nanosheets have a BET surface area of 78.76 m^2^ g^−1^ and a total pore volume of 0.15 cm^3^ g^−1^. The distribution curve of the pore sizes in Fig. S1d† shows that the RSRZ nanosheets have a narrow pore size distribution around 3 nm, indicating mesoporous properties of the as-obtained RSRZ nanosheets.

To investigate the chemical composition, it is necessary to proceed with elemental analysis. Energy dispersive spectroscopy (EDS) elemental mapping images (Fig. 1f) of the RSRZ nanosheets obtained after acidification for 10 h confirmed that Se occupied a majority of the elemental composition, which still contained a fraction of Zn elements. In addition, the FTIR spectra (Fig. S1e, ESI†) confirmed that there was no organic DETA in the RSRZ nanosheets at all after 10 hours of acidification, suggesting that the removal of the amine from the ZnSe(DETA)_0.5_ hybrid precursors was carried out very thoroughly. The peaks in the FTIR near 3430 and 1630 cm^−1^ could be attributed to the stretching and bending vibrations of –OH from absorbed water, indicating oxygen or water adsorption on the surface of the as-obtained RSRZ nanosheets. Raman spectra (ESI, Fig. S1f†) obtained for the RSRZ nanosheets generated from acidification for 10 h showed a sharp peak at 255 cm^−1^, which corresponds to disordered chain-like Se molecules^32–35^ or monoclinic selenium.^36^ The weak and broad peak near 495 cm^−1^ in the Raman spectra demonstrated that the RSRZ nanosheets after acidification for 10 h still contained a fraction of ZnSe.^37^ Hence, it was difficult to define the novel sheets as a single phase. We call the intermediates “red Se remaining Zn” (RSRZ) considering their colour (Fig. 1b) and composition.

H^+^ is so small that it can diffuse into a lamellar structure and attack amines in the ZnSe(DETA)_0.5_ nanosheets. So the pH value is especially important for the reaction system. As a result, the pH was varied from 0.1 to 1 at the same concentration of precursor (see Experimental section 2). TEM and SEM images of the RSRZ nanosheets formed under different pH conditions are shown in Fig. S2(a and b) for pH 0.1, Fig. S2(c and d)† for pH 0.5, and Fig. 1b, c and e for pH 1, respectively. As these images showed, the sheets obtained with pH 0.1 were the most broken. This indicated that the acidity contributes to the porosity of the RSRZ nanosheets. TEM images (Fig. S2(e and f), ESI†) demonstrated that feeding with oxygen or a long acidification time contributed to forming large particles. A higher concentration of precursor, higher temperature and constant stirring would result in nanoframes (Fig. S2g, ESI†). In addition, the phase transformation of the ZnSe(DETA)_0.5_ hybrid during the acidification process was studied using XRD (Fig. S2h, ESI†). The broad peaks of the RSRZ nanosheets after acidification for 2 h could be indexed as hexagonal ZnSe (JCPDS: 15-0105). With prolonging of the reaction time, the RSRZ sheets were gradually acidified by hydrochloric acid and post-oxidized into Se by dissolved oxygen in the solution. The XRD peaks of the sample after acidification for 24 h can be indexed as t-Se (JCPDS: 01-0853).

The aforementioned characterization of the acidification product of ZnSe(DETA)_0.5_ with reaction time showed that the hybrid became a metastable ZnSe phase first and then a stable Se phase by oxidation with oxygen in water, but the details of the transformation of the hybrid into metastable ZnSe were still unclear. To get a better understanding of the stability and reactivity of the ZnSe(DETA)_0.5_ lamellar hybrid during the acidification process, we explored the phase transformation mechanism by tracking the acidification process at time intervals (see Experimental section 3).

**Fig. 2:**
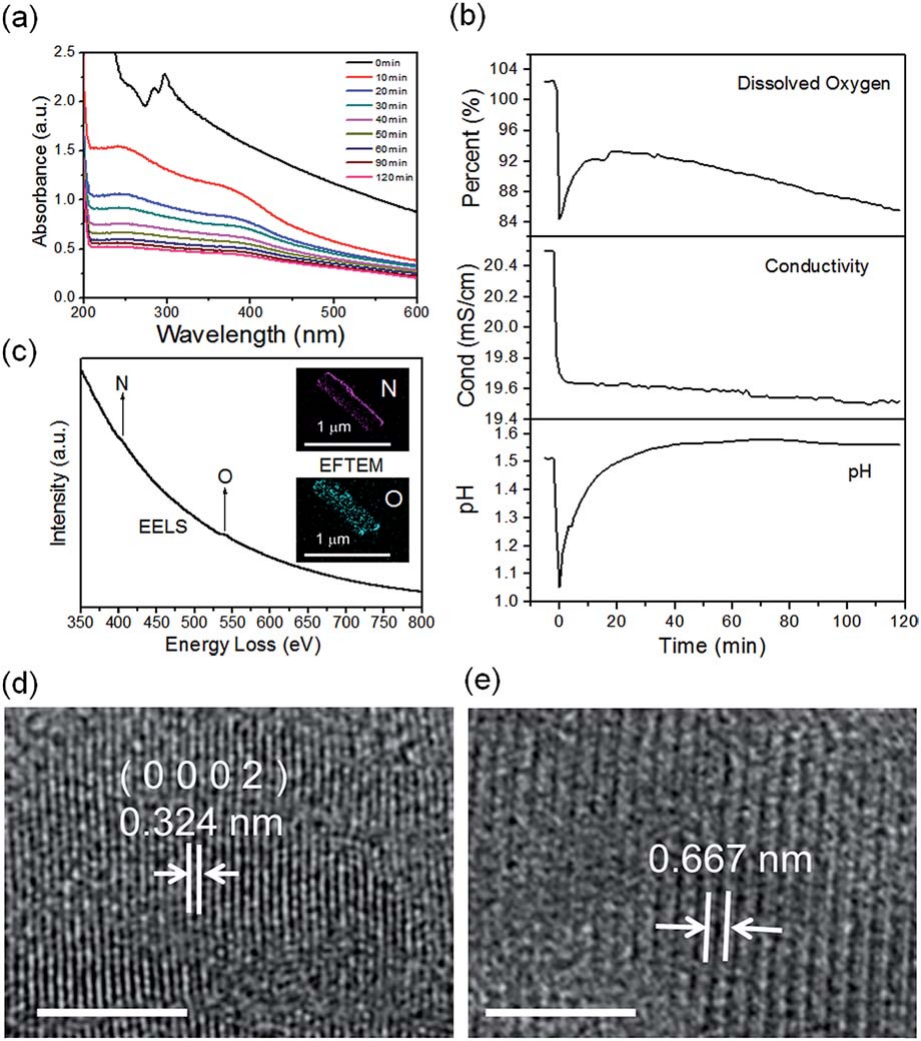
(a) Real-time UV-vis spectra during the reaction at 30 °C; (b) dissolved oxygen, conductivity and pH values during the acidification process. (c) EELS curve and EFTEM images of the sample captured at 10 min during the acidification process. (d and e) HRTEM images of the captured intermediates from the acidification process for 10 min and 120 min, respectively. Scale bar: 5 nm.

The real-time changes of the UV-vis absorption spectrum, the amount of oxygen, the conductivity and the pH value of the reaction system at 30 °C are summarized in Fig. 2a and b. The UV-vis absorption spectrum at 0 min in Fig. 2a is exactly consistent with the previously reported results for ZnSe(DETA)_0.5_.^26^ It is obvious that the peak position of the UV-vis absorption spectrum changed significantly from 0 min to 10 min, indicating that the inorganic–organic hybrid structure of ZnSe(DETA)_0.5_ was destroyed under the acidic conditions. The broad peak near 250 nm in the UV-vis spectrum for the sample obtained after acidification for 10 min matched with the bandgap of ZnSe.^5,38^ In addition, electron energy loss spectroscopy (EELS) was used to analyse the N and O elements of the sample after acidification for 10 min. The smooth curve in Fig. 2c demonstrates that there were hardly any N and O elements in the sheet. It was observed that just a little amount of N elements was distributed only on the edge of the sheet from the energy filtered transmission electron microscopy (EFTEM) images (Fig. 2c). It was also apparent that the content of the O element was larger than the N element using the EFTEM images. As we know, the O element could only occur from adsorption because the ZnSe(DETA)_0.5_ itself did not contain O elements. The small quantity of O elements existing in the sheet may come from absorbed O_2_ or H_2_O. These results illustrated that almost all DETA (C_4_H_13_N_3_) inside the hybrid sheet was successfully depleted and diffused into solution after acidification for 10 min. In other words, the ZnSe(DETA)_0.5_ inorganic–organic hybrid was transformed into inorganic ZnSe. As the reaction proceeded with time, the peak near 250 nm became weaker and weaker, suggesting that more and more inorganic ZnSe was destroyed. This indicated that oxidation had happened. After addition (at 0 min in Fig. 2b) of the hybrid precursors into water which was preheated and adjusted to a desirable pH value in advance, the dissolved oxygen, conductivity and pH of the solution showed an extreme change in the first few minutes because it took a few minutes to mix the solution homogeneously. Then, the dissolved oxygen amount went down slowly because of the consumption of oxygen due to oxidation in the solution being faster than diffusion of oxygen from the air. The phenomenon that the conductivity went down and the pH value went up slowly accounts for consumption of H^+^. All of these proved that oxidation of ZnSe occurred during the acidification process. Thus, the mechanism of the whole acidification included two stages: H^+^ attack on DETA (exfoliation) and oxidation. The related chemical reactions and apparent rate equation for each stage are shown below (the details for all the equations are shown in the ESI†):

Stage 1:1
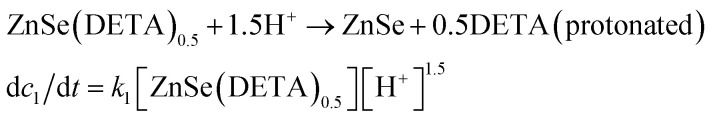


Stage 2:2
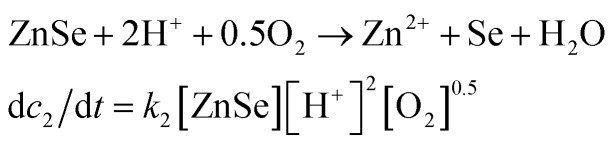

3 *k* = *A* exp^(−*E*_a_/*RT*)^

4 ln *x*_O_2__ = *A* + *B*/T*, T* = *T*/100 K

5 *P*_O_2__ = H*x*_O_2__

To further validate the mechanism, we compared the HRTEM images of a sample obtained after acidification for 120 min with that for 10 min (Fig. 2d and e and S3, ESI†). The crystal lattices corresponded to hexagonal ZnSe (0002) planes for the sample obtained after 10 min (the molar ratio of Zn : Se is 56 : 44 based on EDS elemental mapping analysis shown in Fig. S4a, ESI†). The lattice fringes of the sample after acidification for 120 min became so ambiguous that two lattices combined together, showing an average spacing of 0.667 nm. This means that the crystal structure had been destroyed to some degree during the acidification from 10 min to 120 min, owing to oxidation of ZnSe. Furthermore, real-time tracking of the acidification reaction at different temperatures was carried out (Fig. S4(c and d), ESI†). The process of exfoliation at 50 °C lasted only one minute, but it extended to ten minutes at 10 °C. On the contrary, the process of oxidation at 50 °C was much slower than that at 10 °C. The reason for this is that a high temperature is favourable for increase of the rate constant *k* but also lower oxygen levels in solution. According to eqn (1) and (2), we might draw the conclusion that the reaction rate of stage 1 went up while that of stage 2 went down at high temperature.

As is known, the redox potential is the criterion for a redox reaction to occur. The oxidation of ZnSe at stage 2 could be divided into three steps as shown in eqn (6)–(8). Using eqn (4) and (5), the molar concentration of dissolved oxygen in water was 2.36 × 10^−4^ mol L^−1^ when the temperature was 30 °C (303 K). Thus, the potential of stage 2 in solution was approximately 1.7 V according to the Nernst equation (eqn (9)) when the pH was 1.5 and the molar concentration of Se^2−^ was 
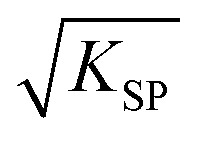
, indicating that the redox reaction would proceed quite easily and thoroughly. Moreover, a gas–solid reaction was extremely likely to happen in the acidic fluid medium. The stabilizer-depleted ZnSe layers were far from the equilibrium state and tended to absorb oxygen and release Zn^2+^, considering that they were ultrathin and porous. All of this means that the captured intermediates during the acidification process were always obtained along with poor stability and high reactivity.

6 ZnSe ↔ Zn^2+^ + Se^2−^, *K*_SP_ = 3.6 × 10^−26^

7 0.5O_2_ + 2H^+^ + 2e^−^ ↔ H_2_O, *E*_θ_ = 1.229 V

8 Se + 2e^−^ ↔ Se^2−^, *E*_θ_ = −0.924 V

9
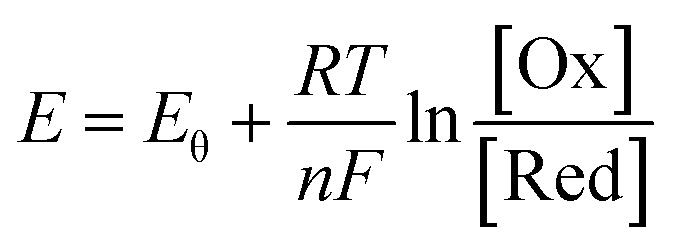


**Fig. 3:**
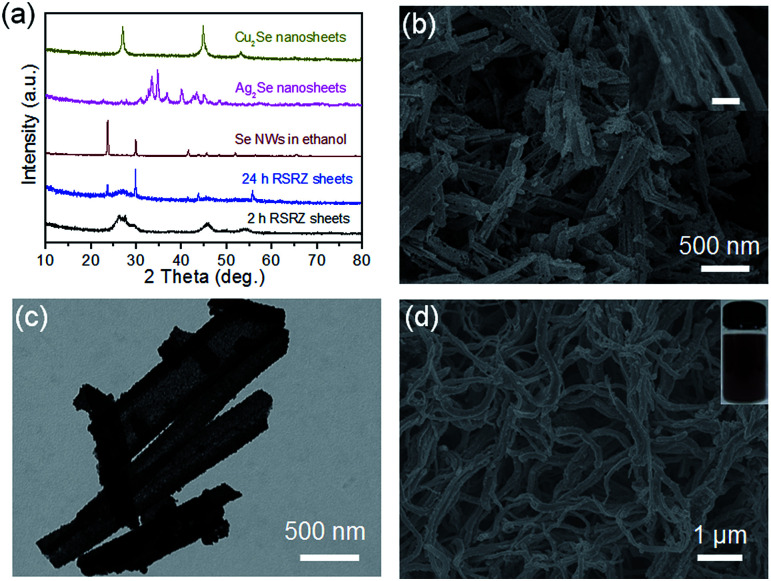
(a) XRD patterns of the RSRZ nanosheets formed from different acidification times (2 h, 24 h), Se nanowires, Ag_2_Se nanosheets, and Cu_2_Se nanosheets. (b) SEM images of the Ag_2_Se nanosheets. Inset scale bar: 100 nm. (c) TEM image of the Pt_3_Se_2_ alloy nanosheets. (d) SEM image of the Se nanowires transformed from the RSRZ nanosheets and a photograph of the corresponding ethanol dispersion after sonication.

Interestingly, the RSRZ sheets have a high chemical activity under ambient conditions making them suitable as a new platform for the synthesis of 2D metal selenides through a facile chemical transformation while maintaining the 2D structures. The RSRZ nanosheets can be easily transformed into Ag_2_Se and Cu_2_Se nanosheets through a chemical transformation process by simply adding AgNO_3_ or CuCl into a suspension containing the RSRZ nanosheets. The XRD patterns of the chemical transformation products are shown in Fig. 3a, which could be indexed as β-Ag_2_Se (JCPDS no. 71-2410) and cubic Cu_2_Se (JCPDS no. 88-2043), respectively. SEM images of the as-synthesized Ag_2_Se and Cu_2_Se are shown in Fig. 3b and S5a,† respectively. Compared to the RSRZ nanosheets, the pore size of the Ag_2_Se and Cu_2_Se nanosheets became much larger, as indicated by the SEM images. In addition, many particles were attached on the surface of the Cu_2_Se nanosheets, which might be due to an Ostwald ripening process during the reaction.

The unique properties and exorbitant price of noble metals like Pt draw intensive attention to Pt-based heteronanostructures or alloy catalysts.^39–41^ Xia’s group reported that hollow nanostructures of Pt could be synthesized by templating with Se nanowires and colloids in 2003.^42^ The RSRZ nanosheets reported here can certainly be used for templating the synthesis of Pt_*x*_Se_*y*_ and Pd_*x*_Se_*y*_ alloys, which is different from the Se@Pt reported previously.^33^

**Fig. 4:**
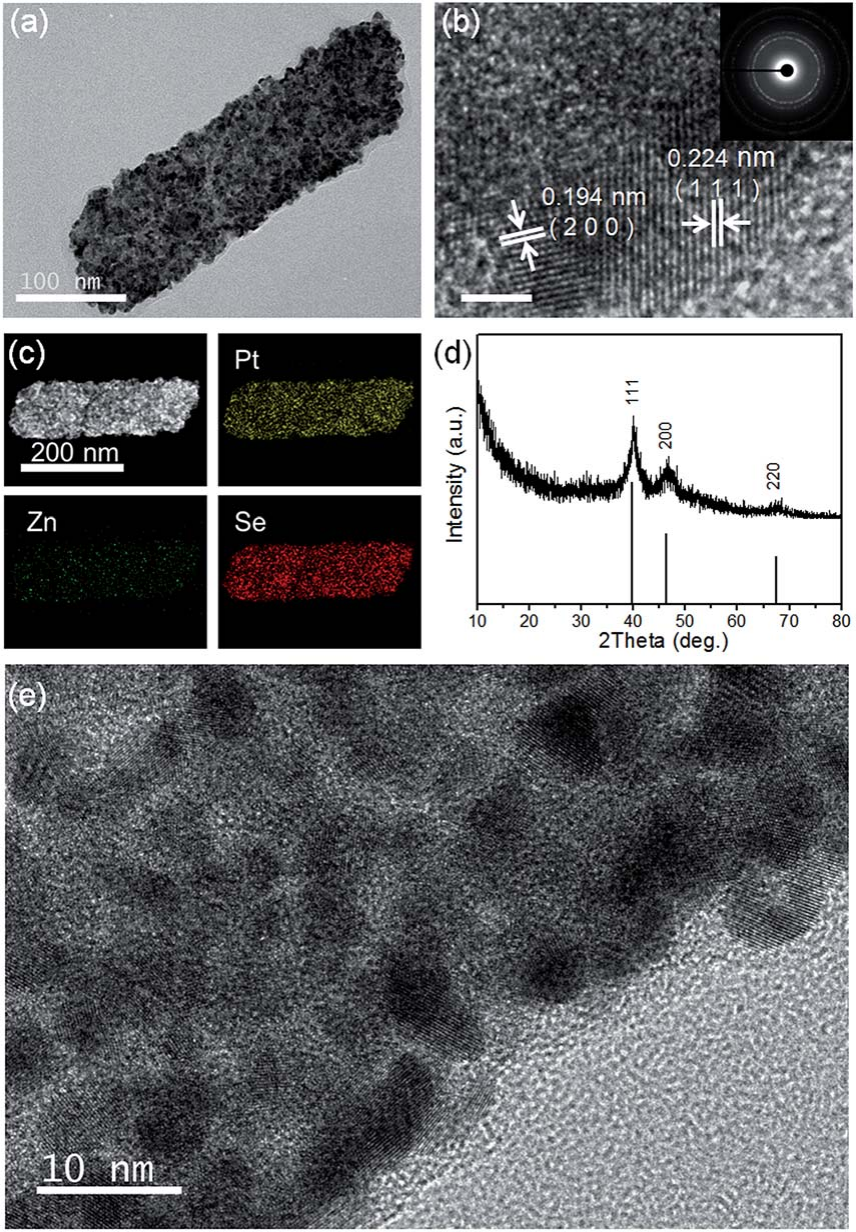
(a) TEM image of the Pt_3_Se_2_ alloy nanosheets; (b) HRTEM image of the Pt_3_Se_2_ alloy nanosheets and the corresponding fast Fourier transform image, scale bar, 2 nm; (c) EDS elemental mapping images of the Pt_3_Se_2_ alloy nanosheets; (d) XRD pattern of the Pt_3_Se_2_ alloy nanosheets; (e) HRTEM image of a Pt_3_Se_2_ alloy nanosheet.

TEM, HRTEM and SAED images of the Pt_*x*_Se_*y*_ alloy nanosheets are shown in Fig. 3c and 4a, b and e. Obviously, the Pt_*x*_Se_*y*_ alloy nanosheets were quite rough, porous and polycrystalline. The lattice spacings of 0.224 nm and 0.194 nm are close to the (111) and (200) facets of Pt (JCPDS # 04-0802), which is consistent with the results from X-ray diffraction (Fig. 4d). EDS elemental mapping analysis (Fig. 4c) demonstrated that Pt and Se elements were uniformly distributed in a single nanosheet, but these nanosheets still contained a tiny minority of Zn elements. The accurate chemical composition was analyzed using inductively coupled plasma (ICP) emission spectroscopy. As a result, the composition was Pt_3_Se_2_, only containing 0.25% Zn. Pt 4f XPS spectra (Fig. S5b, ESI†) indicated that most of the Pt was in the metallic state. This template synthesis could also be applied to Pd, and the related details are shown in the ESI (Fig. S5(c–f) and S6(d and f)†). The difference was that the Pd_63_Se_37_ alloy nanosheets were amorphous and some large particles were attached to the sheets.

In addition, the RSRZ nanosheets can also be transformed into t-Se nanowires by naturally ageing in ethanol. Fig. 3d shows a SEM image of the Se nanowires transformed from RSRZ nanosheets by ageing for two days. The diffraction peaks of the Se nanowires in Fig. 3a can be indexed to trigonal selenium (t-Se, JCPDS # 01-0853). The as-prepared t-Se nanowires may also act as a good template for the synthesis of a variety of nanowires.^42–44^

Amorphous Se tends to form larger colloids.^36,43,45^ Many large particles were observed in our experiment if the RSRZ nanosheets were oxidized more thoroughly (Fig. S2(e and f), ESI†). In particular, the amorphous Se was unstable in alcohol whereas the follow-up transformation would be carried out in a solution containing alcohol. However, the formation of large particles was not desired. Thus, it is better to transform the Se into M_*x*_Se_*y*_ as soon as the nanosheets are partly oxidized to Se, because the remaining ZnSe would be oxidized in the follow-up transformation by keeping the reaction system acidic in the presence of air. Almost all nanomaterials are usually far from the equilibrium state on account of the increased Gibbs free energy.^46^ Poor stability and high reactivity were always found together as a double-edged sword for nanomaterials as we demonstrated previously in the case of ultrathin tellurium nanowires in solution.^47^ The conditions for the formation of different target nanostructures by the acidification of a ZnSe(DETA)_0.5_ hybrid are summarized in Table S1 (ESI).†

## Conclusions

In summary, we have proposed a general route for the synthesis of a family of metal chalcogenide nanostructures by chemical transformation of an intermediate precursor so-called “red Se remaining Zn” (RSRZ) formed through an acidification process of a ZnSe(DETA)_0.5_ lamellar hybrid. The reaction mechanism for transforming such an inorganic–organic hybrid precursor to RSRZ nanosheets has been investigated by tracking the dynamic acidification process. Such RSRZ nanosheets show high chemical reactivity and can serve as a new precursor for the synthesis of various metal selenide nanostructures including Ag_2_Se nanosheets, Cu_2_Se nanosheets, Pt_*x*_Se_*y*_ alloy nanosheets, Pd_*x*_Se_*y*_ alloy nanosheets, and Se nanowires under ambient conditions by chemical transformation, templating synthesis, and structure transformation. The present work demonstrates that having an appropriate balance between the poor stability and high reactivity of a specific layered nanostructure allows it to be possible to capture the highly reactive intermediates, which will provide a new platform for the synthesis of various mesoporous metal selenides.

## Experimental section

Na_2_SeO_3_, diethylenetriamine (DETA), hydrazine hydrate (85%), aqueous ammonia solution (25–28%), HCl (36–38%), ethylene glycol (EG), ethanol, dimethylsulfoxide (DMSO), AgNO_3_, CuCl, PtCl_2_, and PdCl_2_ were purchased from Shanghai Chemical Reagents Co. Ltd. All the chemical reagents were used as received without further purification.

### Synthesis of ZnSe(DETA)_0.5_ inorganic–organic hybrid nanosheets

For this study, we prepared ZnSe(DETA)_0.5_ nanosheets using the reported modified method^31^ based on our first report.^26^ In a typical procedure, Zn(OAC)_2_·2H_2_O (3 mmol) and Na_2_SeO_3_ (3 mmol) were dissolved in a mixed solvent of *V*_N_2_H_4_·H_2_O_/*V*_DETA_/*V*_H_2_O_ = 5 : 14 : 16 to form a homogenous solution under constant strong stirring. The mixed solution was then transferred into a 50 ml Teflon-lined autoclave (with a filling ratio of 80%). The sealed vessel was then maintained at 140 °C for 12 h, and allowed to cool down naturally. The samples were collected and washed three times with water.

### Synthesis of “red Se remaining Zn” nanosheets

The prepared ZnSe(DETA)_0.5_ nanosheets (∼0.075 mmol, 1 ml of the mixed liquid in a Teflon-lined autoclave) were centrifuged and washed three times with water. The precipitate was dispersed in deionized water (40 ml) in a conical flask (volume 50 ml) under constant strong magnetic stirring or sonication. When the solution was homogeneous, commercial hydrochloric acid was added into the conical flask. The pH was adjusted to the required value within a range from 0.1 to 1. The solution was kept under strong magnetic stirring or sonication for 10–20 min. Stirring was stopped once flavescent floccules appeared. Then, the solution was kept standing for a few minutes and 3/4 of the supernate was poured out. At last, the conical flask was kept open and left standing for 1 to 24 h under air. The colour changed obviously: from milk white to yellow, then red. Finally, the red precipitate washed with HCl and water for characterization. The experiment was conducted at room temperature. It is easy to scale up (we have tried for 20 times the scale, Fig. S4f†).

### Dynamic acidification process

#### The reaction system

The prepared ZnSe(DETA)_0.5_ nanosheets (∼0.15 mmol, 2 ml of the mixed liquid in Teflon-lined autoclave) were centrifuged and washed three times with water. Then, deionized water (200 ml) was poured into the specific container under constant strong stirring. 1 ml of HCl was dropped into the container. The prepared ZnSe(DETA)_0.5_ nanosheets (∼0.15 mmol) dispersed in deionized water (20 ml) were added into the container. We took 2 ml samples into cuvettes for obtaining the UV-vis absorption spectra every few minutes. The whole process was performed under constant strong magnetic stirring. A constant temperature was controlled using a precise low temperature thermostat.

#### The experimental apparatus

The real-time measurements of dissolved oxygen, pH and conductivity during the experiment were carried out using a digital precision meter Multi 9430 (WTW GmbH., Germany) (ref. 47).

### Follow-up transformations based on the RSRZ nanosheets

Table S1 in ESI† shows the appropriate conditions used for the RSRZ nanosheets from Experimental section 2 for the different transformations.

#### Synthesis of Se nanowires

The prepared red precipitate was dispersed in ethanol (20 ml) in a sample bottle. The bottle was placed in a dark corner for natural sedimentation for various amounts of time. Finally, the grey precipitate was washed with HCl, water and ethanol for characterization. The experiment was conducted at room temperature.

#### Synthesis of Ag_2_Se nanosheets

The prepared red precipitate was dispersed in 35 ml of EG in a conical flask (volume 50 ml) under constant strong stirring for 10 min. Then, 0.1 g of AgNO_3_ (dispersed in 5 ml of DIW) was dropped into the conical flask with vigorous magnetic stirring until there was a lot of black precipitate on the bottom. The reaction was very fast. The experiment was conducted at room temperature.

#### Synthesis of Cu_2_Se nanosheets

The prepared red precipitate was dispersed in 35 ml of EG (ethylene glycol) in a conical flask (volume 50 ml) under constant strong stirring for 10 min. Then, 0.05 g of CuCl (dispersed in NH_3_·H_2_O) was added into the conical flask with vigorous magnetic stirring for 40 min until the colour become brown. The experiment was conducted at room temperature.

#### Synthesis of Pt and Pd nanosheets

The solution in Experimental section 2 containing an orange precipitate was dispersed in 30 ml of ethanol or EG under constant strong stirring for 10 min. Then, 40 mg of PtCl_2_ or 30 mg of PdCl_2_ (dispersed in HCl solution) was added into the mixed solution. The mixed solution was shaken at a rotation rate of 260 rpm using an Innova 40 Benchtop Incubator Shaker for 10 h at 60 °C. The products were collected by centrifugation (5000 rpm, 5 min) and washed with DMSO (to remove PtCl_2_), water and ethanol.

### Characterization

Scanning electron microscopy (SEM) images were obtained with a Zeiss Supra 40 scanning electron microscope at an acceleration voltage of 5 kV. The transmission electron microscopy (TEM) and high-resolution transmission electron microscopy (HRTEM) observations were performed with a Hitachi H-7650 microscope at 100 kV and a JEOL ARM-200F transmission electron microscope at 200 kV, respectively. Energy dispersive spectroscopy (EDS), electron energy loss spectroscopy (EELS) and energy filtered transmission electron microscopy (EFTEM) were carried out on a JEOL ARM-200F transmission electron microscope. XRD patterns were recorded on a PW1710 instrument with CuKα radiation *λ* = 0.15406 nm. XPS spectra were obtained with an ESCALab MKII X-ray photoelectron spectrometer using a Mg Kα radiation excitation source. UV-vis spectra were recorded on a UV-2501PC/2550 spectrometer (Shimadzu Corp., Japan) at room temperature. The infrared spectra were measured on a NICOLET Fourier transform infrared spectrometer, using pressed KBr tablets. The atomic force microscopy study in the present work was performed using a Veeco DI Nano-scope MultiMode V system. The BET measurements were determined using Micromeritics ASAP-2000 nitrogen adsorption apparatus. Raman spectra were obtained with a confocal laser microRaman spectrometer (LABRAM-HR, JY Co.).

## Supplementary Material

SC-007-C6SC00674D-s001
