# Supplementary material for: A general chemical transformation route to two-dimensional mesoporous metal selenide nanomaterials by acidification of a ZnSe–amine lamellar hybrid at room temperature
Source: Chem Sci. 2016 Mar 9;7(7):4276–83. doi: 10.1039/c6sc00674d (PMC6013810; doi:10.1039/c6sc00674d)
Supplement: SC-007-C6SC00674D-s001 [file SC-007-C6SC00674D-s001.pdf]

Electronic Supplementary Material (ESI) for Chemical Science.
This journal is © The Royal Society of Chemistry 2016

Electronic Supplementary Material (ESI) for Chemical Science.
This journal is © The Royal Society of Chemistry 2014

Supplementary Information

A general chemical transformation route to two-dimensional mesoporous metal selenide nanomaterials by acidification of ZnSe-Amine lamellar hybrid at room temperature †

Zeng-Wen Hu,‡ Liang Xu,‡ Yuan Yang, Hong-Bin Yao, Hong-Wu Zhu, Bi-Cheng Hu, and Shu-Hong Yu*

Division of Nanomaterials & Chemistry, Hefei National Laboratory for Physical Sciences at the Microscale, Collaborative Innovation Center of Suzhou Nano Science and Technology, Department of Chemistry, CAS Center for Excellence in Nanoscience, Hefei Science Center of CAS, University of Science and Technology of China, Hefei 230026, China
E-mail: shyu@ustc.edu.cn

*Corresponding authors: shyu@ustc.edu.cn

Notes of equations in the article:

Eqs. (1) and (2) are apparent kinetic equation. Eq. (3) is Arrhenius equation. Eqs (4), (6), (7) and (8) refer to CRC Handbook of Chemistry & Physics. Eq. (5) is Henry's law and H is Henry constant. Eq. (9) is Nernst equation. k is the apparent rate constant for equation (1), (2) and (3). [ ] is the molar concentration for Eqs. (1), (2) and (9). x is the molar fraction for equation (4) and (5). For Eq. (4), When P_{O_2} = 101.3 kPa, A= -66.7, B = 87.5, C = 24.5.

 This journal is © The Royal Society of Chemistry [year]

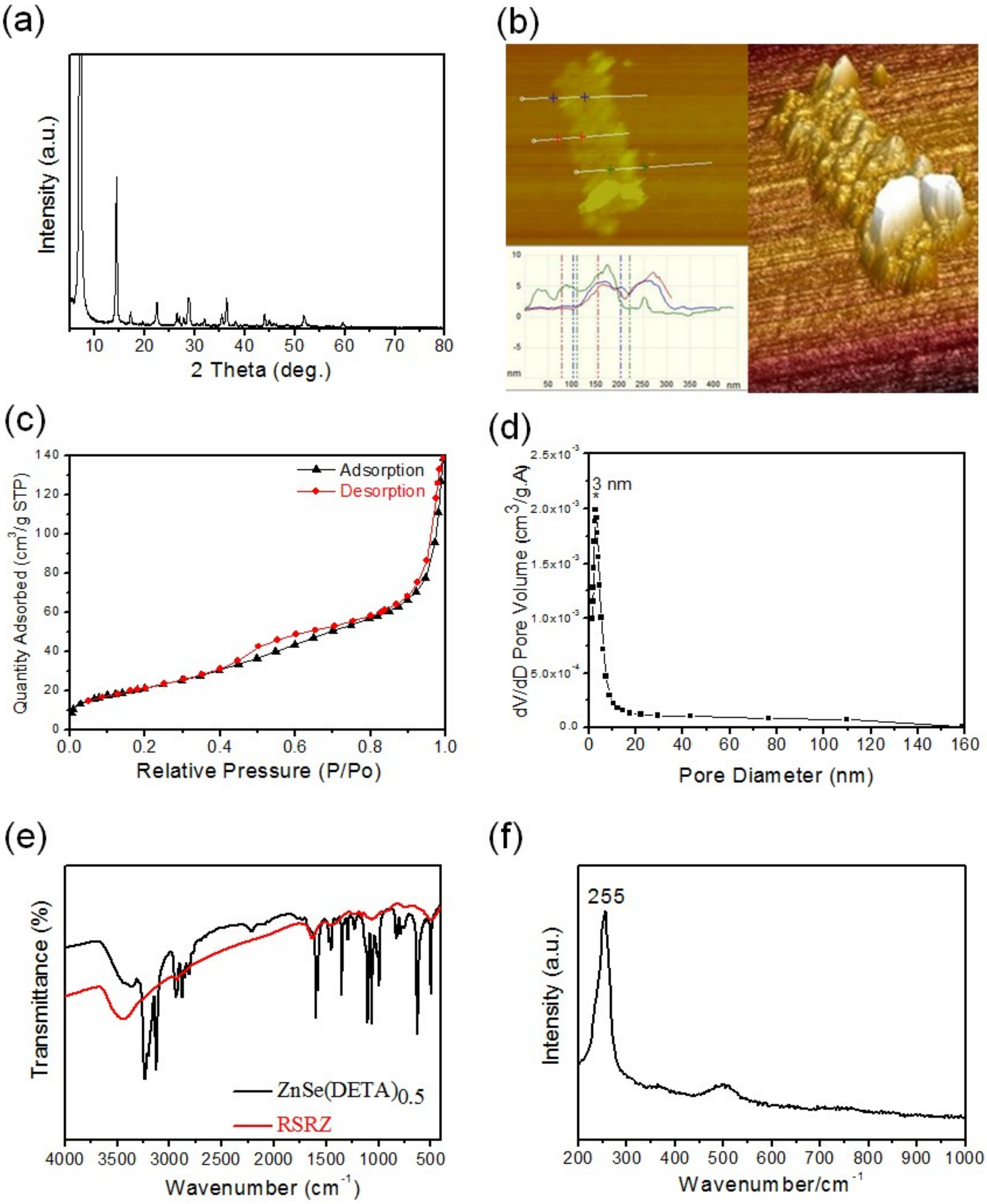

Fig. S1 (a) XRD pattern of $ZnSe(DETA)_{0.5}$ nanosheets. (b) AFM images (3D) and the associated height profiles of the RSRZ nanosheets obtained after acidification for 10 h. (c) Nitrogen adsorption–desorption isotherms of the RSRZ nanosheets obtained after acidification for 2 h. (d) BJH adsorption dV/dD pore volume of the RSRZ nanosheets obtained after acidification for 2 h. (e) FTIR spectra of $ZnSe(DETA)_{0.5}$ and the RSRZ nanosheets obtained after acidification for 10 h; (f) Raman spectra of the RSRZ nanosheets obtained after acidification for 10 h. Notes: we chose the RSRZ nanosheets obtained after acidification for 2 h rather than 10 h for BET test because Se nano* was hard to bear high temperature during desorption.

This journal is © The Royal Society of Chemistry [year]

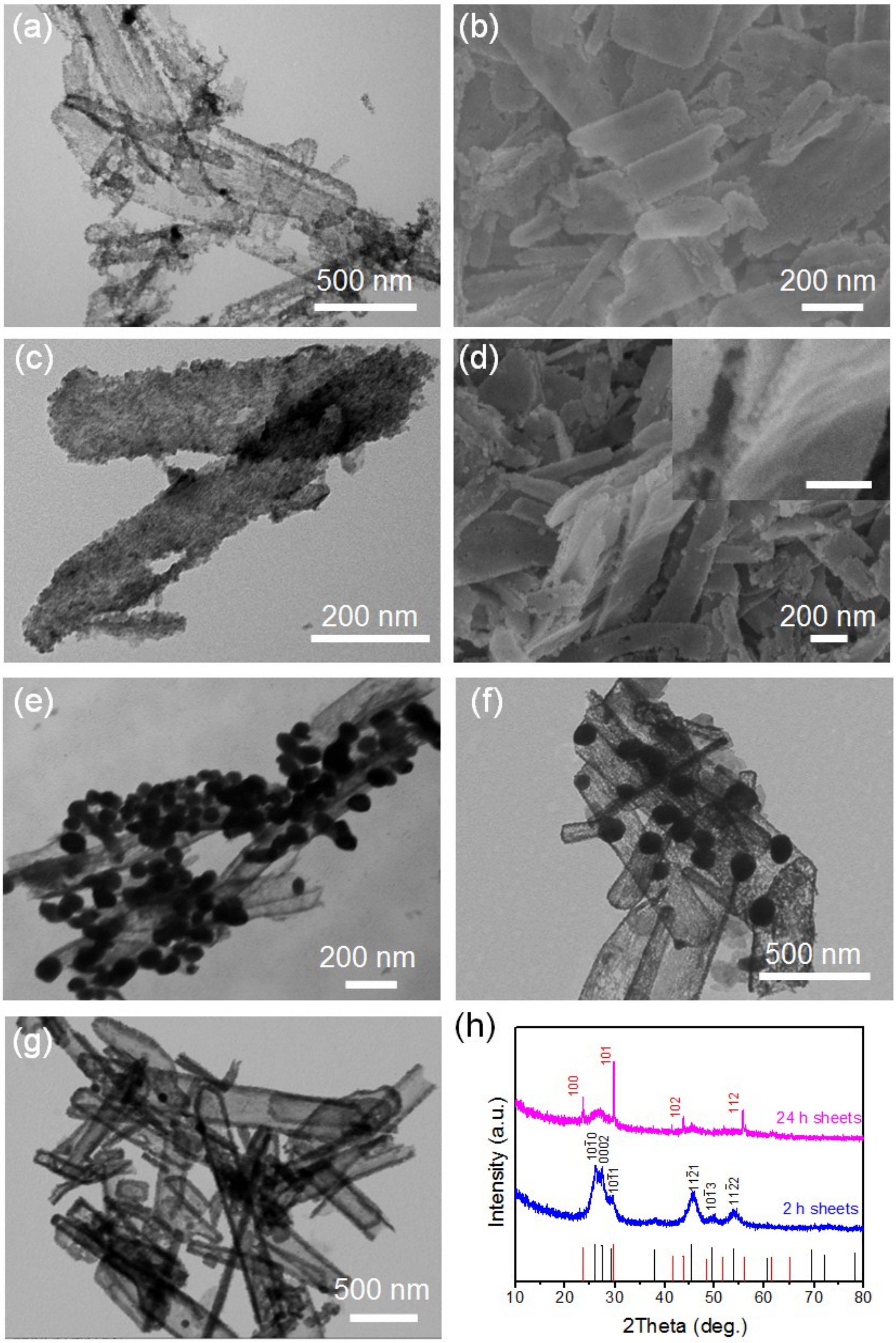

Fig. S2 (a, b) TEM and SEM images of the RSRZ nanosheets (pH 0.1). (c, d) TEM and SEM images of the RSRZ nanosheets (pH 0.5). Inset scale bar: 100 nm. (e) TEM image of the RSRZ nanosheets by feeding oxygen. (f) TEM image of RSRZ nanosheets kept for 24 h in acid solution. (g) TEM image of the RSRZ nanoframes. (h) XRD patterns of the RSRZ sheets formed in different acidification time (2 h, 24 h).

This journal is © The Royal Society of Chemistry [year]

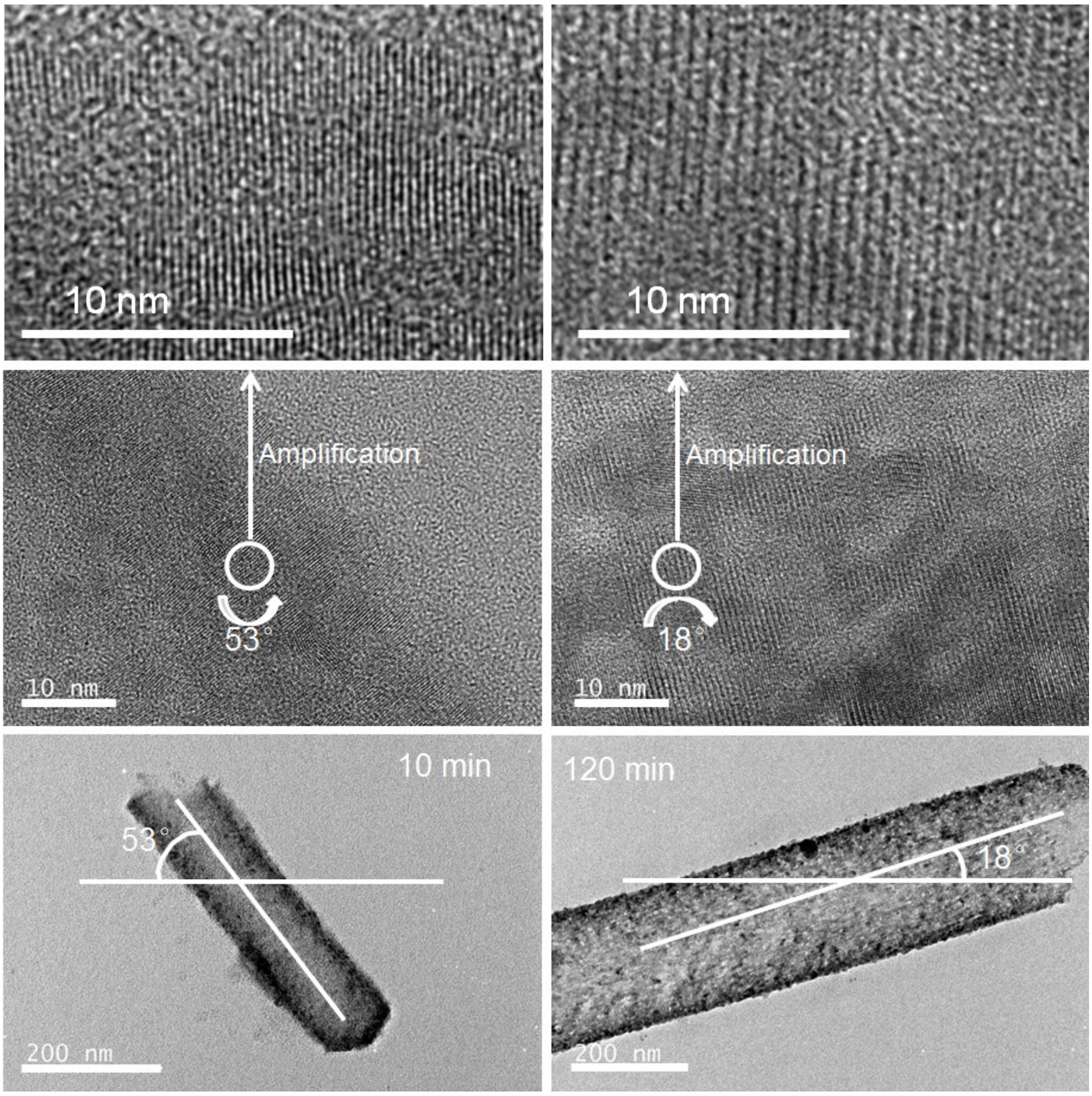

Fig. S3 HRTEM analysis for the samples shown in Fig. 2d and Fig. 2e, respectively.

This journal is © The Royal Society of Chemistry [year]

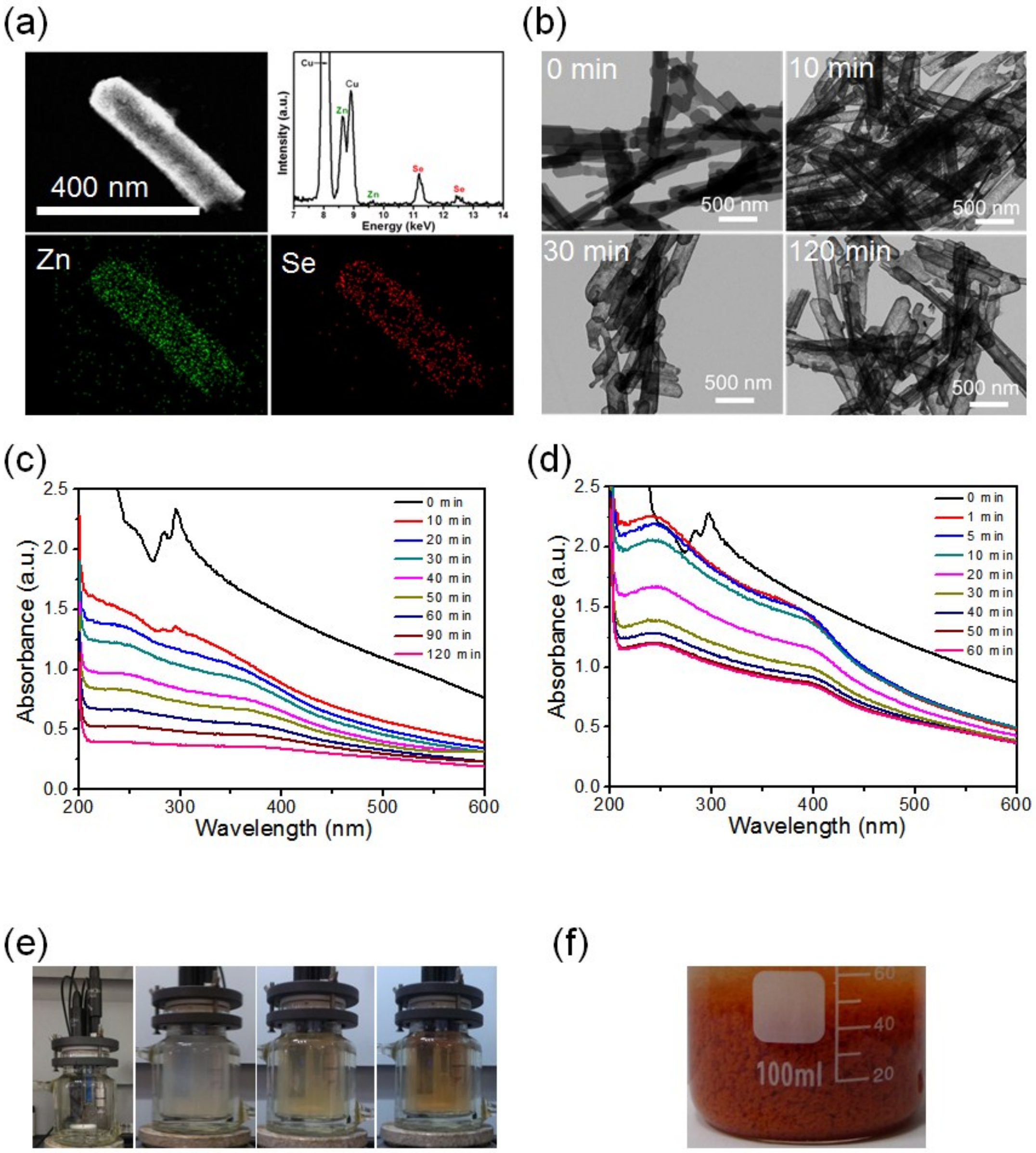

Fig. S4 (a) EDS elemental mapping images of the sample after acidification for 10 min, corresponding to the sample for the UV–Vis absorption spectra at 30 ºC. (b) TEM images of the samples after acidification for 0 min, 10 min, 30 min and 120 min, respectively, corresponding to the samples for the UV–Vis absorption spectra at 30 ºC. (c, d) UV-vis spectra of the dynamic state during the acidification process at 10 ºC and 50 ºC. (e) The experimental device and color variations of experiment section 3. (f) Photograph of the RSRZ nanosheets in water prepared as descripted in Experiment Section 2 (The yield was scaled up to 20 times).

This journal is © The Royal Society of Chemistry [year]

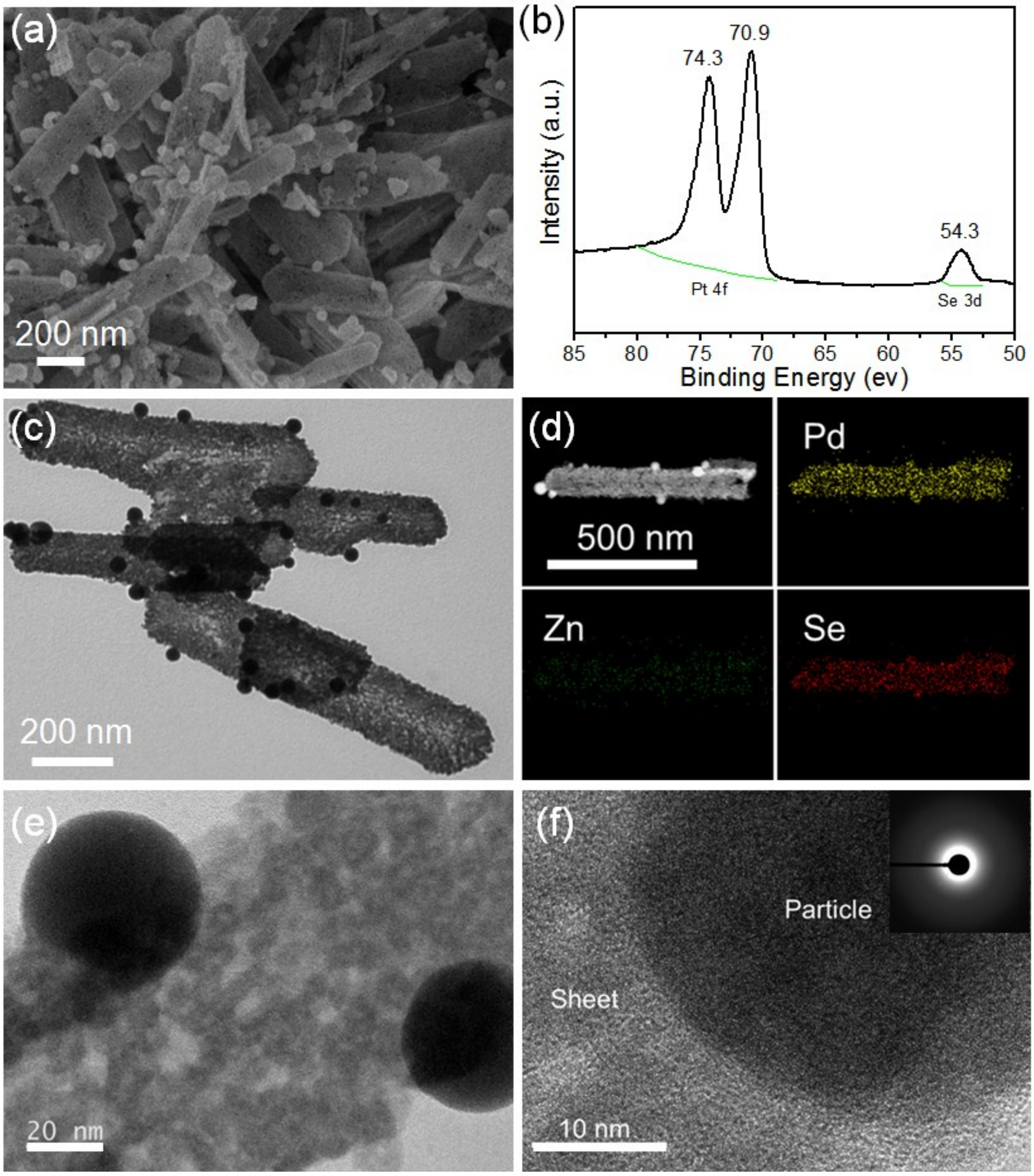

Fig. S5 (a) SEM image of Cu_2Se nanosheets. (b) XPS spectrum of Pt_3Se_2 alloy nanosheets. (c) TEM image of $Pd_{63}Se_{37}$ alloy nanosheets. (d) EDS elemental mapping images of $Pd_{63}Se_{37}$ alloy nanosheets. (e, f) HRTEM images of $Pd_{63}Se_{37}$ alloy nanosheets and the corresponding fast Fourier transform.

This journal is © The Royal Society of Chemistry [year]

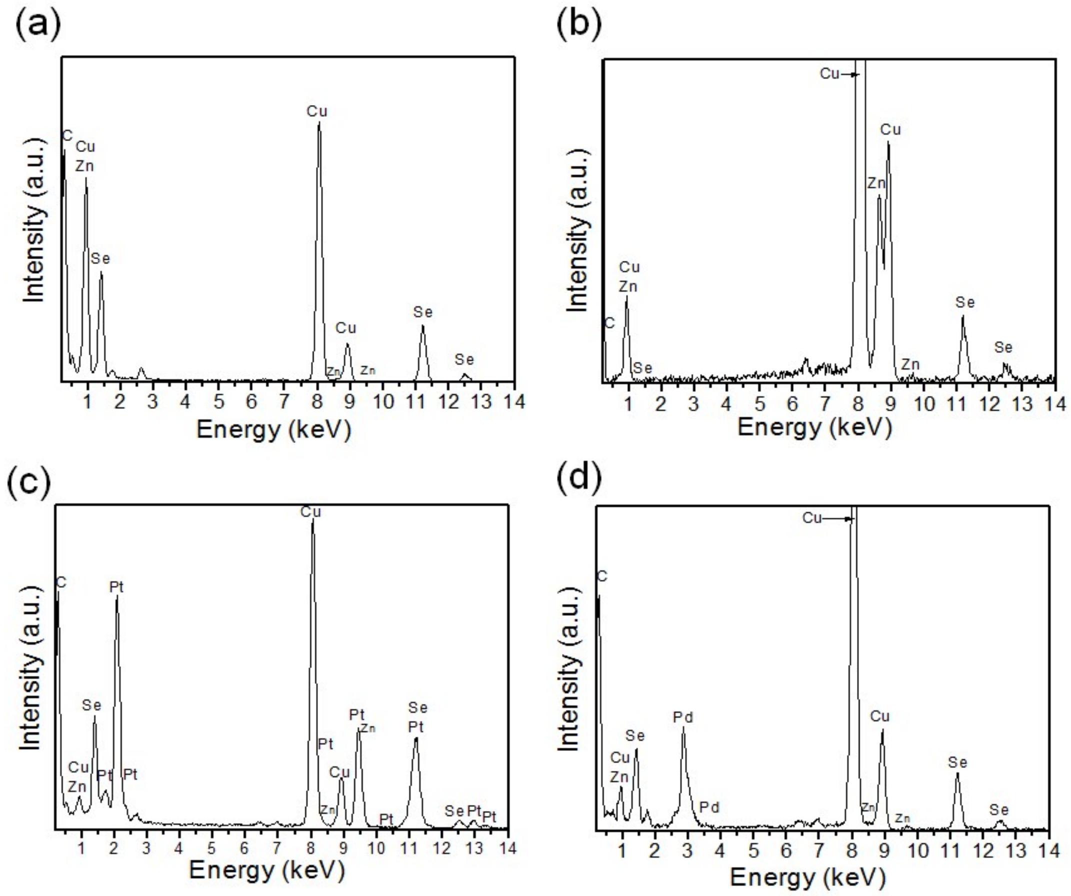

(e)

Element	EDS	ICP
Zn	-5.36%	0.25%
Se	39.01%	40.38%
Pt	66.35%	59.37%

(f)

Element	EDS	ICP
Zn	-10.54%	0.23%
Se	54.29%	36.58%
Pd	56.25%	63.19%

Fig. S6 (a, b, c, d) The EDS spectra for element mapping images of Fig. 1f, Fig. S4a†, Fig.4c and Fig. S5d†. (e, f) Composition of Pt_3Se_2 and $Pd_{63}Se_{37}$ alloy nanosheets. Notes: element Cu and C came from copper screen used for TEM. The atomic percent of Zn was negative through EDS analysis because there was hardly Zn in the sample and the value which was outputted by the computer of TEM instrument was automatically dealt with deducting background.

 This journal is © The Royal Society of Chemistry [year]

Table S1 Conditions of the captured RSRZ nanosheets prepared as described in Experimental Section 2 for different transformation process described in Experimental Section 4.

Target	pH	Time	Pretreatment	Colour
For Se NWs	0.1	> 4 h	Washing with water and ethanol	Red
For Ag_2Se and Cu_2Se NSs	1	4-6 h	Washing with water	Red
For Pt and Pd NSs	1	2-4 h	No washing	Orange

Notes: orange colour meant that ZnSe occupied in the majority of composition.

Synthesis of RSRZ nanoframes in Fig. S2g†. The prepared $ZnSe(DETA)_{0.5}$ nanosheets (~0.15 mmol, 2 ml of the mixed liquid in Teflon-lined autoclave) were centrifuged and washed three times with water. The precipitate was dispersed into deionized water (40 ml) in a conical flask (volume 50 ml) under constant strong magnetic stirring. When the solution was homogeneous, 2 ml commercial hydrochloric acid was added into the conical flask. The solution was kept strong magnetic stirring for 40 min. Then, the solution was kept standing for a few minutes and 3/4 of supernate was poured out. At last, the conical flask was kept open standing in the air till floccule became red. The experiment was operated at 40 ^{o}C.

This journal is © The Royal Society of Chemistry [year]

Acidification of ZnS-Amine

Similar acidification could apply to ZnS-Amine. Fig. S7a† and Fig. S7e† showed that $ZnS(DETA)_{0.5}$ hybrid nanosheets were synthesized successfully. After the acidification of $ZnS(DETA)_{0.5}$ hybrid precursors, mesoporous ZnS nanosheets (Fig. S7b†, Fig. S7c†, Fig. S8d† and Fig. S7e†) were obtained. It was a little different from the acidification of $ZnSe(DETA)_{0.5}$ that $ZnS(DETA)_{0.5}$ hybrid was hard to bear strong acidity because ZnS trended to dissolve in acid solution. In addition, the obtained mesoporous ZnS nanosheets were not easy to be oxidized.

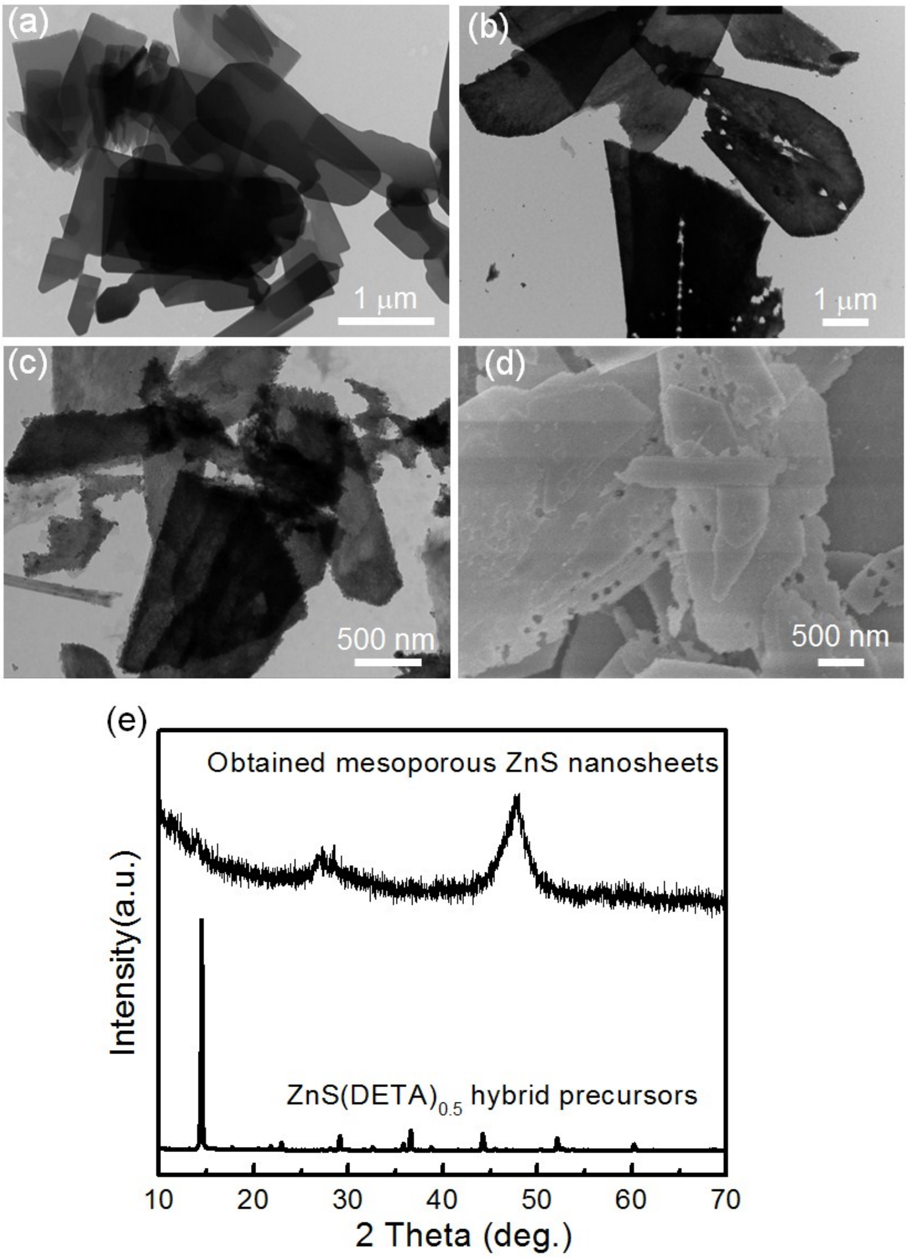

Fig. S7 (a) TEM image of $ZnS(DETA)_{0.5}$ nanosheets. (b, c) TEM images of mesoporous ZnS nanosheets. (d) SEM image of mesoporous ZnS nanosheets. (e) XRD patterns of $ZnS(DETA)_{0.5}$ precursors and mesoporous ZnS nanosheets after acidification.

 This journal is © The Royal Society of Chemistry [year]

Synthesis of $ZnS(DETA)_{0.5}$ hybrid nanosheets. Here we prepared $ZnS(DETA)_{0.5}$ nanosheets using a modified method based on the previous report.[1, 2] In a typical procedure, $Zn(NO_3)_2 \cdot 6H_2O$ (3 mmol) and thiourea $CS(NH_2)_2$ (3 mmol) were dissolved into a mixed solvent of DETA:H_2O = 8:1 to form a homogenous solution under constant strong stirring. The mixed solution was then transfered into a 50 ml Teflon-lined autoclave (with a filling ratio of 80%). The sealed vessel was then maintained at 180 °C for 10 h, and cooled down naturally. The samples were collected and washed three times with water.

Acidification of $ZnS(DETA)_{0.5}$ hybrid nanosheets. The prepared $ZnS(DETA)_{0.5}$ nanosheets (2 ml of the mixed liquid in Teflon-lined autoclave) were centrifuged and washed three times with water. The precipitate was dispersed into deionized water (40 ml) in a conical flask (volume 50 ml) under constant strong magnetic stirring or sonicating. When the solution was homogeneous, 0.04 ml of commercial hydrochloric acid was added into the conical flask (PH ≈ 2.2). The solution was kept strong magnetic stirring for 20 min. Then, the solution was kept standing for 24 h. Finally, the white precipitate washed water for characterization. The experiment was operated at room temperature.

Notes and references

1. W. T. Yao, S. H. Yu, L. Pan, J. Li, Q. S. Wu, L. Zhang and J. Jiang, *Small*, 2005, **1**, 320-325.
2. Y. Yu, J. Zhang, X. Wu, W. Zhao and B. Zhang, *Angew. Chem. Int. Ed.*, 2012, **51**, 897-900.

This journal is © The Royal Society of Chemistry [year]
